# Appendiceal Gastrointestinal Stromal Tumor: A Case Report and Review of the Relevant Literature

**DOI:** 10.7759/cureus.105831

**Published:** 2026-03-25

**Authors:** Toshimitsu Maki, Takahisa Fujikawa, Ippei Yamana, Izumi Kinoshita, Suguru Hasegawa

**Affiliations:** 1 Surgery, Kokura Memorial Hospital, Kitakyushu, JPN; 2 Surgery, Fukuoka University Chikushi Hospital, Fukuoka, JPN; 3 Pathology, Kokura Memorial Hospital, Kitakyushu, JPN; 4 Gastroenterological Surgery, Fukuoka University Chikushi Hospital, Fukuoka, JPN

**Keywords:** appendix, gastrointestinal stromal tumor, gastrointestinal surgery, laparoscopic surgery, pathological findings

## Abstract

Gastrointestinal stromal tumor (GIST) is the most common type of mesenchymal tumor found in the gastrointestinal tract, but appendiceal GIST is very rare. It is often discovered when patients present symptoms that resemble those of acute appendicitis. We herein report the case of an 81-year-old man who presented with abdominal pain. He underwent a laparoscopic appendicectomy with histopathological examination confirming a completely resected GIST of the appendix. GIST of the appendix is rare, with only a few reported cases. We currently present a case of appendiceal GIST that was diagnosed postoperatively. Preoperative diagnosis of appendiceal GIST is difficult because of its small size. Therefore, surgeons must be cautious when considering the possibility of an appendiceal GIST and ensure adequate surgical margins.

## Introduction

Gastrointestinal stromal tumors (GISTs) account for 0.2-0.5% of all gastrointestinal tumors and are the most common mesenchymal tumors originating from the digestive tract [1]. The most frequent site for GISTs is the stomach (60%), followed by the small intestine (30%) and colon (5% to 15%) [1]. The occurrence of GISTs originating from the greater omentum, mesentery, and esophagus is very low. Appendiceal GIST is very rare, constituting approximate 0.1% of all GIST [1]. Appendiceal GISTs are misdiagnosed as acute appendicitis and treated with appendectomy, as they can present with appendicitis-like symptoms. Differential diagnoses to consider are appendiceal adenocarcinoma [2], appendiceal neuroendocrine tumor [3], and appendiceal diverticulitis [4]. Furthermore, it can be discovered incidentally during imaging, during surgery for other diseases, or at autopsy. We herein present a case of an appendiceal GIST that was resected laparoscopically.

## Case presentation

**Figure 1:**
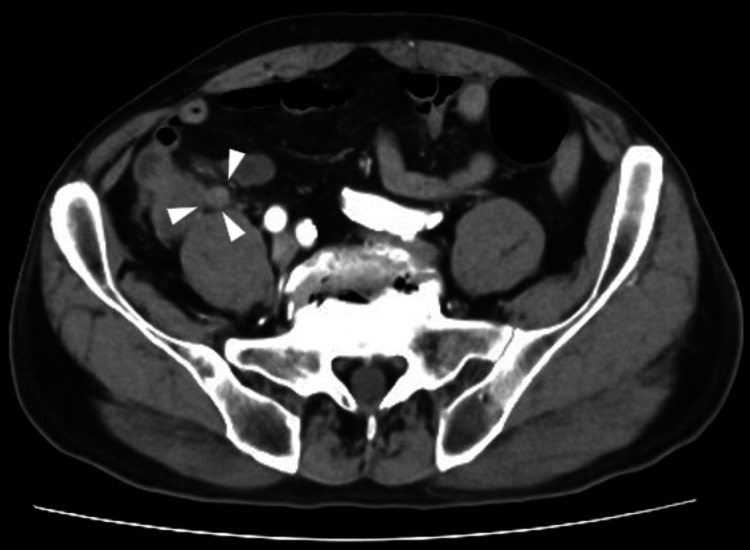
Abdominal CT The CT scan revealed inflammatory changes in the appendix (arrow).

An 81-year-old man presented with abdominal pain and a significant medical history, including a cerebrovascular accident, hypothyroidism, hypertension, and sigmoid colon cancer surgery. He was diagnosed with acute appendicitis and treated conservatively. However, one month later, the patient experienced a recurrence of abdominal pain. Laboratory tests showed a WBC count of 6,400/μL and a CRP level of 0.8 mg/dL. Abdominal contrast-enhanced computed tomography revealed an enlarged appendix with wall enhancement and increased density in the surrounding fatty tissue; however, no abscess formation was observed (Figure 1). Based on these findings, the patient was diagnosed with chronic appendicitis and underwent a laparoscopic appendectomy.

**Figure 2:**
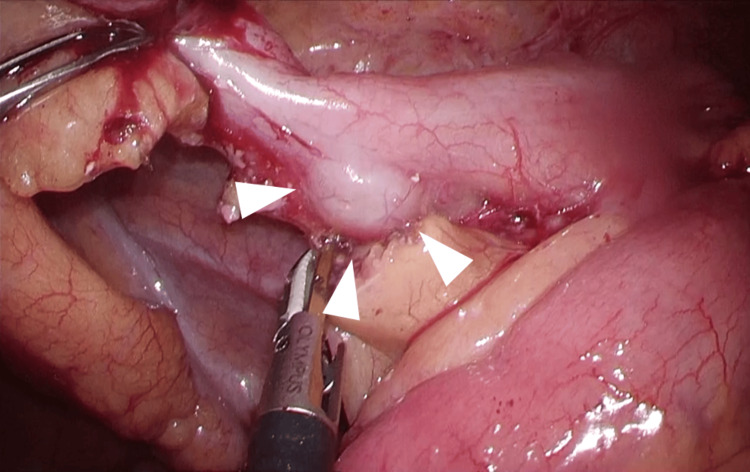
Intraoperative findings The appendix was enlarged, and a 10 mm mass was found at a site proximal to the appendix (arrow).

Intraoperative findings revealed swelling of the appendix in the right lower abdomen. No ascites was found in the abdominal cavity. A mass measuring approximately 10 mm was observed near the proximal side of the appendix (Figure 2). The appendix was transected, ensuring that the tumor was on the resected side, after double ligation of the intact proximal side with ENDOLOOP™ Ligature (Johnson & Johnson MedTech, USA). The operative time was 46 min, and the blood loss was 5 ml.

**Figure 3:**
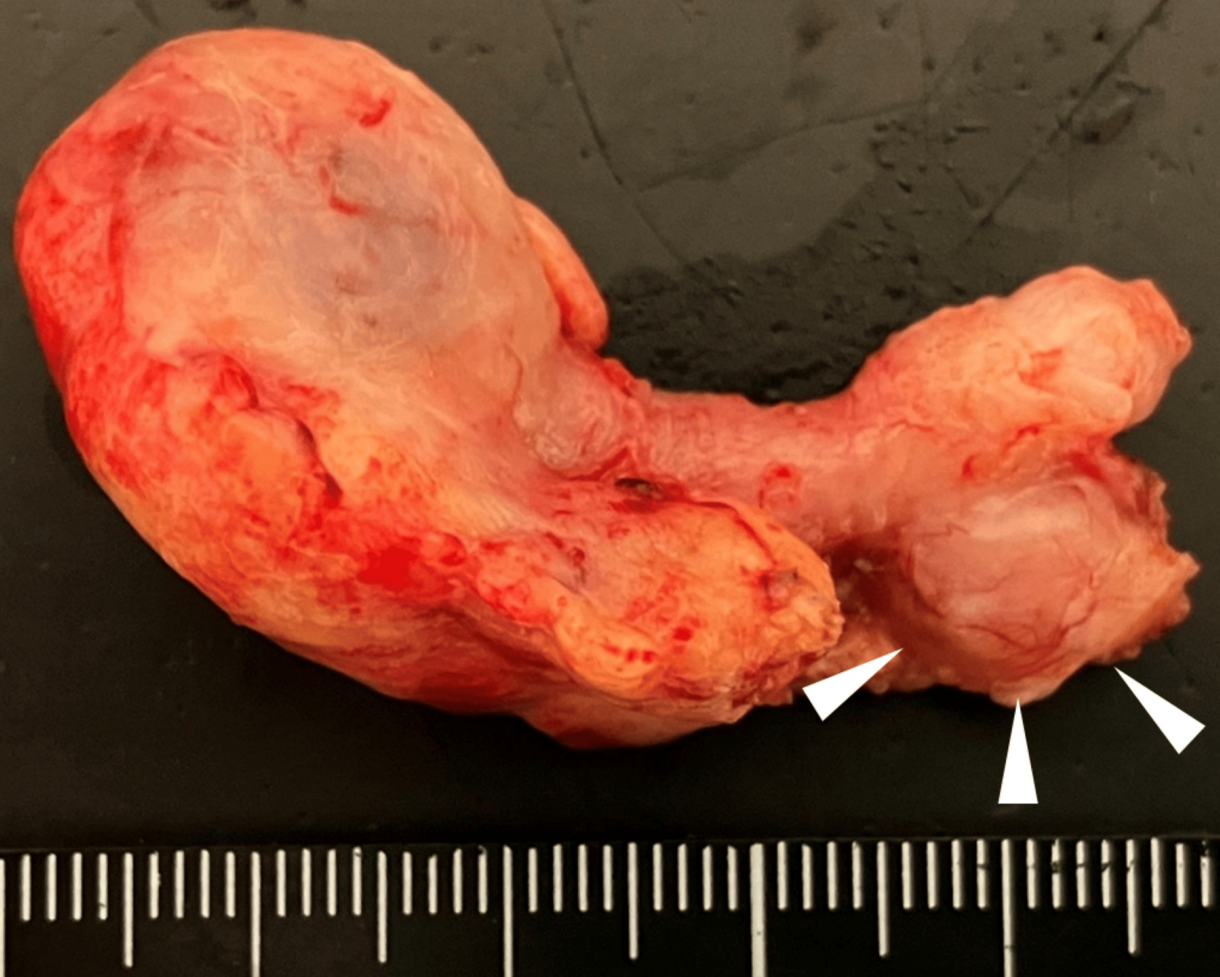
Surgical specimen The resected specimen revealed a mass measuring 10 mm in diameter in the proximal appendix (arrow).

**Figure 4:**
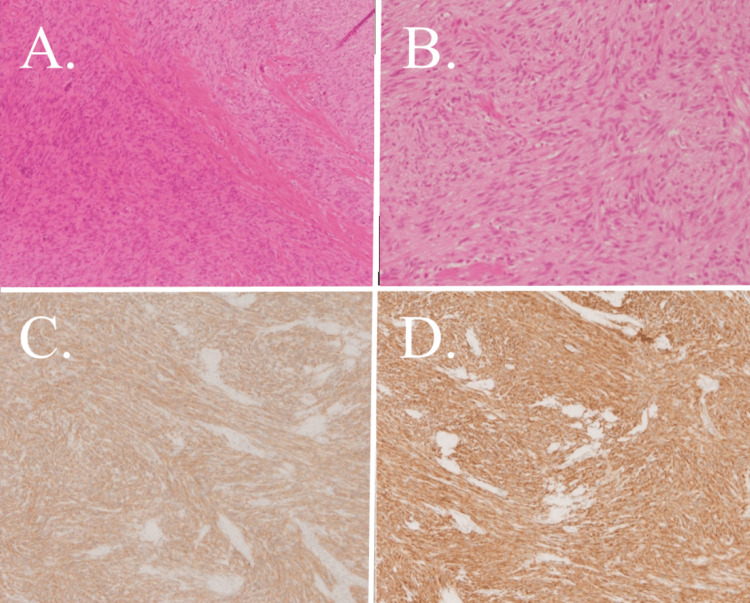
Histopathological findings (A) A hematoxylin and eosin (H&E)-stained section at 100× magnification. Spindle-shaped cells with oval or elliptical nuclei proliferated in a fascicular or complex pattern. (B) An H&E-stained section at 200× magnification. There was no nuclear atypia, and mitotic activity was absent. (C) A CD117-stained section at 100× magnification. The tumor cells were positive for CD117 (c-kit). (D) A CD34-stained specimen section at 100× magnification. Tumor cells were CD34 positive.

In the resected specimen, the appendix was 50 mm long and 8 mm in diameter, and had a mass measuring 10 mm in diameter at a site proximal to the appendix (Figure 3). Histologically, the tumor was composed of spindle cells, which is characteristic of GIST, with irregular fascicles. There was no nuclear atypia, and mitotic activity was absent (<1/50/high-power field). Immunohistochemistry showed the strong expression of CD117 (c-kit) and CD34 in tumor cells. No reactivity was observed for smooth muscle actin, desmin, or S100protein (Figure 4). The risk of recurrence, according to the Modified Fletcher Classification, was very low.

The patient experienced no postoperative complications and was discharged six days after surgery. One year has passed since the surgery, and he remains under outpatient follow-up with no findings suggestive of recurrence or metastasis.

## Discussion

GISTs are mesenchymal tumors that arise in the gastrointestinal tract and the mesentery. They are defined as tumors that express KIT or CD34 and originate from or differentiate into interstitial cells of Cajal located within the muscularis propria. A GIST is most commonly found in the stomach and small intestine, whereas primary appendiceal GIST is very rare.

A search for "GIST" and "appendix" in PubMed revealed only 15 articles [5-18]. Table 1 summarizes the characteristics of the 19 cases, including our own. The median age of the patients (15 men and four women) was 71 years (range: 37-86). Tumor locations included the proximal part of the appendix in six cases, the middle part in six, and the distal part in four (three were unknown). Appendiceal GIST is smaller than that found in other organs [1]. The median tumor size in the reported cases was 13 mm (range: 0.5-220 mm). Our case's tumor was also small at 13 mm. Eleven patients were diagnosed with symptoms resembling appendicitis, and one presented as an abdominal mass, while the remaining seven had no symptoms attributable to appendiceal GIST. In only two cases, a preoperative diagnosis of GIST was made thanks to their large size, and biopsy confirmed the diagnosis. Twelve patients underwent appendectomy for appendicitis. An appendectomy was performed in two cases due to the identification of an appendiceal abnormality during surgeries on other organs. In two cases, appendiceal GISTs were incidentally found in specimens after right hemicolectomy was performed for other diseases, and one patient was diagnosed at autopsy.

**Table 1:** Summary of the 19 reported cases with appendiceal GIST. GIST, gastrointestinal stromal tumor; M, male; F, female; NA, not available; VLR, very low risk; LR, low risk; HR, high risk; INT, intermediate risk.

No.	Authors	Year	Age (y)	Sex	Symptoms	Site	Size (mm)	Treatment	Risk
1	Miettinen et al. [5]	2002	63	M	None	Distal	14	None	VLR
2	Miettinen et al. [5]	2002	56	M	Appendicitis-like	Proximal	12	Appendectomy	VLR
3	Miettinen et al. [5]	2002	59	M	None	Middle	9	Appendectomy	VLR
4	Miettinen et al. [5]	2002	72	M	Appendicitis-like	Proximal	13	Appendectomy	VLR
5	Yap et al. [6]	2005	66	M	Appendicitis-like	Middle	2.5	Appendectomy	NA
6	Kim et al. [7]	2007	56	M	None	Middle	NA	Right hemicolectomy	NA
7	Rahimi et al. [8]	2008	65	F	None	NA	11	Right hemicolectomy	VLR
8	Agaimy et al. [9]	2008	86	F	None	NA	0.5	NA	VLR
9	Agaimy et al. [10]	2008	73	F	Appendicitis-like	Proximal	5	NA	VLR
10	Agaimy et al. [10]	2008	72	M	None	Distal	25	Appendectomy	LR
11	Elazary et al. [11]	2010	71	M	Appendicitis-like	Distal	200	Appendectomy, Short bowel resection	HR
12	Chung et al. [12]	2012	67	M	Appendicitis-like	Middle	60	Right hemicolectomy	INT
13	Boussadida et al. [13]	2013	75	M	Appendicitis-like	Middle	20	Appendectomy	NA
14	Tran et al. [14]	2014	74	F	Appendicitis-like	Proximal	5	Appendectomy	NA
15	Chun et al. [15]	2016	60	M	Appendicitis-like	Proximal	30	Appendectomy	LR
16	Kaneko et al. [16]	2017	71	M	Abdominal tumor	Distal	220	Imatinib, Appendectomy	HR
17	Williams et al. [17]	2024	74	M	None	Middle	15	Appendectomy	LR
18	Alshaikh et al. [18]	2025	37	M	Appendicitis-like	NA	10	Appendectomy	NA
19	Maki et al. (present case)	2025	81	M	Appendicitis-like	Proximal	13	Appendectomy	VLR

Appendiceal GIST is often characterized by low-grade malignancy. Among the reported 11 cases, nine were categorized as low or very low risk, whereas only two were categorized as high risk. Among the two high-risk cases, an unusual case reported by Kaneko et al. featured a 22-cm high-grade malignant appendiceal GIST. In such cases, neoadjuvant chemotherapy with imatinib, a c-KIT tyrosine kinase inhibitor, has been administered to reduce the tumor size and achieve complete en bloc excision.

According to the National Comprehensive Cancer Network and European Society of Medical Oncology guidelines, complete en bloc resection is necessary for resectable tumors. When an appendiceal tumor is suspected before or during surgery, it is necessary to consider the possibility of malignancy, including GIST. We performed the necessary surgical procedure, including securing the margins and preventing perforation. In our case, a preoperative diagnosis was difficult to make. However, the mass was identified during surgery, and en bloc resection was successfully performed.

## Conclusions

We encountered a case of an appendiceal GIST which was successfully resected laparoscopically. Appendiceal GIST is rare and a preoperative diagnosis is difficult to make. We should therefore consider the possibility of GIST when a tumor is incidentally found in the appendix and aim to achieve complete surgical resection.
